# Effects of tannase-converted green tea extract on skeletal muscle development

**DOI:** 10.1186/s12906-020-2827-7

**Published:** 2020-02-11

**Authors:** Ki-Bae Hong, Hee-Seok Lee, Jeong Sup Hong, Dong Hyeon Kim, Joo Myung Moon, Yooheon Park

**Affiliations:** 1 BK21 Plus, College of Health Science, Korea University, Seoul, 02841 Republic of Korea; 2 Department of Food Science and Technology, Chung-Ang University, Anseong, 17546 Republic of Korea; 3 Animal Center and Preclinical Evaluation Research Institute, Yonam College, Cheonan, 31005 Republic of Korea; 4 BTC Corporation, #703, Technology Development Center, 705 Haean-ro, Sangnok-gu, Ansan-si, Gyeonggi-do, Republic of Korea; 5 Department of Food Science and Biotechnology, Dongguk University, Goyang, 10326 Republic of Korea

**Keywords:** Tannase-converted green tea extract, (−)-epicatechin, (−)-epigallocatechin, Skeletal muscle mass, Sarcopenia

## Abstract

**Background:**

The aim of this study was to investigate the effect of tannase-converted green tea extract with a high (−)-epicatechin (EC), (−)-epigallocatechin (EGC), and gallic acid (GA) content on myotube density and fusion in normal and oxidative stress-induced C2C12 skeletal muscle cells. Although the use of green tea extract is considered beneficial, cellular and molecular mechanisms of action of tannase-converted green tea extracts that are used as potential muscle growth materials have not been thoroughly studied.

**Methods:**

This study used histological analysis and molecular biology techniques, and compared the results with those for AMPK activator 5-aminoimidazole-4-carboxamide-1-β-D-ribonucleoside (AICAR) and green tea extracts.

**Results:**

The myotube density of normal and oxidative stress-induced C2C12 cells was significantly higher in the tannase-converted green tea extract-treated group than that observed in the other groups (normal cells: *P* < 0.01; oxidative stress-induced cells: *P* < 0.05). In addition, tannase-converted green tea extract and green tea extract treatments significantly upregulated the genetic expression of myogenin, Myf5, and MyoD (*P* < 0.05). The levels of AMP-activated protein kinase-α (AMPKα) and muscle RING-finger protein-1 (MuRF-1) in the tannase-converted green tea extract group were higher than those in the AICAR and green tea extract groups (*P* < 0.05).

**Conclusions:**

Taken together, our findings describe that the high levels of EC, EGC, and GA in the tannase-converted green tea extract are attributable to the morphological changes in C2C12 cells and intercellular signaling pathways. Therefore, tannase-converted green tea extract can be used in the treatment of sarcopenia.

## Background

Aging is a predominant risk factor for common diseases, and previous studies have focused on the age-related physiological changes occurring in the molecular and cellular mechanisms [1]. In addition, cell senescence is a response to a variety of stressors and is a major target for therapeutic application and antiaging therapy. The loss of skeletal muscle associated with aging causes functional disability due to the loss of strength, risk of falls, fracture, and loss of autonomy [2]. Although the prevalence of sarcopenia is high in individuals whose age is ≥60 years, accounting for 5–13% of all adults [3], the number of patients with sarcopenia is expected to rise as the aging population continues to increase globally. Skeletal muscle is the dominant organ system in locomotion and energy metabolism, and its differentiation, growth and metabolism are regulated by neurotransmitters, hormones, growth factors, cytokines, and nutritional factors [4]. In addition, the induction sarcopenia is involved in one of several underlying mechanisms of major complications, and physical inactivity is known to increase chronic reactive oxygen species (ROS) overproduction during the progression of sarcopenia [5].

Even though the adapted physical activity, vitamin D administration and Mediterranean diet are a possible non-pharmacologic treatment to prevent or treat muscle atrophy [6–8], the development of an effective method for its management and treatment is still required. Recently, the use of botanical extracts and nutraceutical compounds via dietary sources has been focused on, to enhance muscle growth and physiological activity. Several botanicals (citrus, coffee, ginger, ginseng, grape, and turmeric, etc.) are known to have a significant level of activity in the prevention of muscle damage and pain resulting from inflammation and oxidative stress. More recent studies in animal models and in vitro demonstrate the antioxidative and anti-inflammatory roles of nutraceutical compounds including polyphenols, flavonoids, and phenolic acids through the modulation of the levels of proteins, plasma enzymes, cytokines, and receptors related to the immune response [9].

Green tea harvested from *Camellia sinensis* contains polyphenols and it is widely used in nutraceutical and pharmaceutical industries. Diverse studies have been conducted regarding tea production, the extraction process, storage, and optimum conditions [10]. In addition, an impressive number of studies have consistently assessed the role of green tea polyphenols in liver and heart diseases, and different types of methods for cancer prevention [11]. Most of the green tea polyphenols are flavanols, and a plant chemical known as a catechin, which is an antioxidant. The polyphenol components of green tea, such as (−)-epigallocatechin gallate (EGCG), (−)-epicatechin (EC), (−)-epigallocatechin (EGC), and (−)-epicatechin gallate (ECG) are used as anti-inflammatory [12], and anti-oxidative [13].

Several approaches have been extensively applied to improve the total catechin content, function, and pharmacological properties of green tea, and several studies have reported that enzyme hydrolysate enhances total catechins and/or (−)-epicatechin content and biological properties [14]. (−)-Epicatechin has been reported to affect several different signaling pathways by giving rese to differences in tissue specificity, it consists of two aromatic rings linked by an oxygenated heterocycle with a 4-hydroxyl group [15]. Previous studies have revealed that green tea extract played a role in muscle recovery, but the effects of the enzyme-converted green tea catechins on skeletal muscle mass and relevant mechanisms are yet to be studied.

The purpose of this study was to investigate whether the effects of tannase-converted green tea extract with a high EC, EGC, and gallic acid (GA) content on myotube density, fusion, and muscle atrophy are greater than those of green tea extract in normal and oxidative stress-induced C2C12 skeletal muscle cells. Therefore, in this study, results obtained with 5′-AMP-activated protein kinase (AMPK) activator 5-aminoimidazole-4-carboxamide-1-β-D-ribonucleoside (AICAR) and green tea extract were compared using histological analysis and molecular biology techniques. Our findings describe the morphological changes occurring in the C2C12 cell, intercellular signaling pathways associated with sarcopenia, and the therapeutic potential of EC, EGC, and GA obtained from tannase-converted green tea extract.

## Methods

### Materials and chemicals

Tannase-converted green tea extract and green tea extract were obtained from BTC Co. Ltd. (Ansan, South Korea). The green tea extract was hydrolyzed by tannase (Kikkoman Biochemifa, Tokyo, Japan) and obtained the supernatant. The tannase-converted green tea extract was prepared as described in a previous study [16]. HPLC analytical grade standard EGCG, EGC, ECG, EC, GA, and caffeine were purchased from Sigma-Aldrich (St. Louis, MO, USA), and acetic acid and acetonitrile were obtained from Fisher Scientific (Pittsburgh, PA, USA). A specific activator (AICAR) was purchased from Sigma-Aldrich (St. Louis, MO).

### HPLC analysis

The analysis of the catechin content was performed using an HPLC system (Waters e2695 Separations Module, USA) and a UV detection system, as described in previous reports [14]. The HPLC system for catechin, GA, and caffeine measurement used standard materials, and consisted of the Hypersil C18 column (5 μm, 25 × 0.46 cm ID) and a UV-Vis detector. The mobile phase contained 1% acetic acid (solvent A) and acetonitrile (solvent B), with a linear gradient commencing at 92/8 (A/B ratio) and finishing at 73/27 over 40 min, at a flow rate of 1 mL/min.

### Cell cultures

The C2C12 mouse myoblast cell line (ATCC® CRL1772™) was obtained from the American Type Culture Collection (ATCC; Manassas, VA, USA). All cell types were maintained in Dulbecco’s modified Eagle medium (DMEM) containing 10% fetal bovine serum (FBS) and 1% penicillin-streptomycin (10,000 U/mL) at 37 °C in a humidified atmosphere of 5% CO_2_ in air. To induce differentiation in C2C12 cells, 5 × 104 cells were seeded in six-well plates and cultured in growth media until 80–90% confluence was attained. Then, the media were replaced with DMEM media containing 2% horse serum and 1% penicillin-streptomycin (10,000 U/mL). For the cell viability assay, differentiated C2C12 cells were treated with tannase-converted green tea extract (1, 5, 10, 15, and 20 μg/mL) or green tea extract (1, 5, 10, 15, and 20 μg/mL) and cultured for 24 h. A total of 15 μL of 3-(4,5-dimethylthiazol-2-yl)-2,5-diphenyltetrazolium bromide (MTT, Thermo Fisher Scientific, Lombard, IL, USA) was added to each well and incubation was carried out for 3 h. One hundred microliters of DMSO were added to each well and incubation was carried out for 30 min. Absorbance was measured at 560 nm. The relative survival rate of the treated group was calculated based on the survival rate of the normal group that was not treated with 100% of the drug.

### Giemsa staining

The Giemsa staining method of analysis was modified and performed according to the method described by Veliça [17]. C2C12 cells in wells were washed with phosphate-buffered saline (PBS), fixed with 100% methanol for 5 min, and dried for 10 min. The Jenner staining solution (BDH, Poole, UK) was diluted 1:3 in 1 mM sodium phosphate buffer (Sigma-Aldrich, pH 5.6) and incubated for 5 min. After washing with PBS, the wells were incubated with 1 mL Giemsa staining solution (BDH) that was diluted 1:10 times in 1 mM sodium phosphate buffer for 10 min at room temperature. The wells were then washed 2–3 times with PBS and used to analyze the morphological changes in C2C12 cells. The histological indices of C2C12 myogenesis were analyzed, based on the method described by Veliça et al. [17].

### RNA isolation and mRNA expression

The TRIzol® reagent (Invitrogen, CA, USA) was used for total RNA isolation, according to the manufacturer’s protocol. One microgram of total RNA was treated with RQ1 RNase-free DNase I (Promega, WI, USA) and reverse transcribed using SuperScript® III Reverse Transcriptase (Invitrogen), using oligo (dT) primer. Real-time PCR (qRT-PCR) was performed using the Taqman Gene Expression Master Mix (Applied Biosystems, CA, USA), and quantitative analyses were conducted using the StepOne plus Software V. 2.0 (Applied Biosystems). All results were determined based on a validated control gene, 18S RNA, using the ΔΔCt method [18]. Information for target genes used in qRT-PCR is as follows: Myogenin (NM_031189.2), Myf5 (NM_008656.5), MyoD (NM_010866.2), FOXO1 (NM_019739.3), FOXO3 (NM_019740.2), SOD (NM_011434.1), CAT (NM_009804.2), and GST (NM_001251762.2).

### Western Immunoblotting

The cultured cells were washed with PBS 2–3 times, and 150 μL of RIPA Buffer was added. The cells were lysed for 30 min and centrifuged at 12,000×g for 10 min at 4 °C. Protein concentration was quantified using standardizing BSA (bovine serum albumin). Ten μg of lysate was denatured with 10% Mini-protean TGX™ and transferred to a polyvinylidene difluoride (PVDF) membrane at 100 V for 1 h. The membrane was blocked with TBST (0.1% Tween 20 + TBS) solution containing 5% skim milk for 1 h. The primary antibody was diluted with skimmed milk (1:1000) and the reaction was allowed to occur overnight at 4 °C, after which washing was carried out 3 times using TBST. The HRP secondary antibody (horseradish peroxide (HRP) conjugated IgG secondary antibody (Cell Signaling, #5157, 1:2000) was diluted 1: 1000 times, allowed to react for 2 h at 4 °C, washed three times with TBST, and allowed to react with the ECL substrate. Protein levels were detected with a specific antibody, using the ChemiDoc™ imaging systems (Bio-Rad, Hercules, CA).

### Statistical analysis

All analyses were conducted using the R-software (version 3.2.5, The R Foundation, Vienna, Austria). *P*-values were derived from Duncan’s multiple-range test, and a value of *P* < 0.05 was considered to be statistically significant. Values are expressed as the means ± standard deviation (SD) for each group, and all experiments were repeated 4 times.

## Results

### The effects of Tannase-converted green tea extract on C2C12 Myogenesis and muscle regulatory factors

**Fig. 1:**
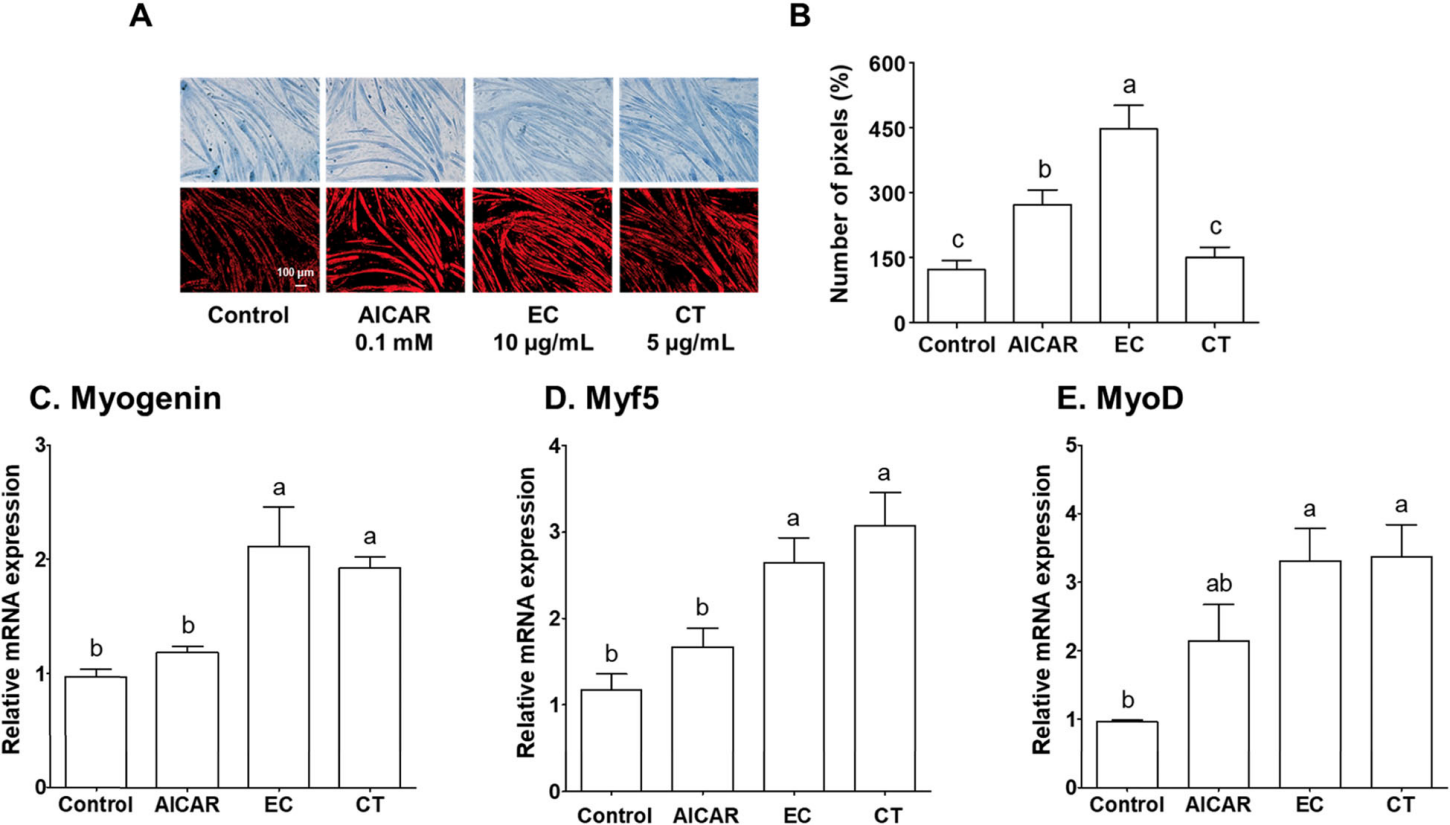
The effects of tannase-treated catechin on (**a** and **b**) myotube formation and the gene expression of (**c**) myogenin, (**d**) Myf5 and (**e**) MyoD in C2C12 skeletal muscle cells. The myotube density was calculated as the sum of pixels attributed to tones 0–75. Each value represents the mean ± SE. Different letters indicate significant differences at *P* < 0.05 according to Tukey’s test. AICAR: AMPK activator 5-aminoimidazole-4-carboxamide-1-β-D-ribonucleoside; EC: tannase-converted green tea extract containing a high epicatechin content; CT: green tea extract

In the present study, the significant difference in total catechin content in tannase-converted green tea extract containing high epicatechin content (EC) and green tea extract (CT) was investigated (Table 1) the changes in C2C12 cell morphology were compared with those observed in AICAR and green tea extract groups (Fig. 1 and Additional file 1). To examine the effects of EC, CGC, and gallic acid (GA) on myogenesis, C2C12 cells were cultured in the presence of 10 μg/mL EC for 12 h and the results were compared with those obtained with the use of 0.1 mM AICAR and 5 μg/mL CT. Solutions with these concentrations were used after a confirmation was obtained through the MTT (3-(4,5-Dimethylthiazol-2-yl)-2,5-diphenyltetrazolium bromide) cell viability assay (data not shown). EC was found to increase myotube density and fusion (Fig. 1a). As shown in Fig. 3-1b, the extent of myotube formation was calculated to quantify the morphological changes, and similar measurements for the myotube density were observed after treatment. The myotube density was significantly higher in the AICAR-treated group (AICAR vs. control, 2.22-fold increase, *P* < 0.05) and the EC group (EC vs. control, 3.66-fold increase, *P* < 0.01).

**Table 1:** Catechin content during tannase treatment

Process	EGCG (mg/g)	EGC (mg/g)	ECG (mg/g)	EC (mg/g)	GA (mg/g)	Caffeine (mg/g)
Raw material	601.02 ± 09.21	185.00 ± 1.2	99.35 ± 0.45	61.34 ± 0.82	1.66 ± 0.29	18.29 ± 0.98
After enzyme reaction	–	511.73 ± 1.8	–	106.20 ± 1.43	209.45 ± 2.12	14. 95 ± 0.85

Values are expressed as the mean ± standard deviation

We examined the mRNA expression of myogenin, Myf5 and MyoD to understand the effects of EC on the mechanism of C2C12 myogenic differentiation. As shown in Fig. 1c-e, EC and CT significantly upregulated the expression of myogenin (EC vs. control, 2.19-fold increase, *P* < 0.05; CT vs. control, 1.99-fold increase, *P* < 0.05), Myf5 (EC vs. control, 2.26-fold increase, *P* < 0.05; CT vs. control, 2.62-fold increase, *P* < 0.05), and MyoD (EC vs. control, 3.43-fold increase, *P* < 0.05; CT vs. control, 3.50-fold increase, *P* < 0.05). However, the the expression of myogenic regulatory factors did not significantly increase in the AICAR-treatment group. This study demonstrated the effects of EC on myogenic genes, such as myogenin, Myf5, and MyoD in C2C12 cells, and showed that EC and CT altered the transcriptional control of gene expression in the skeletal muscles (Fig. 1c-e).

### The effects of Tannase-converted green tea extract on transcription factors

**Fig. 2:**
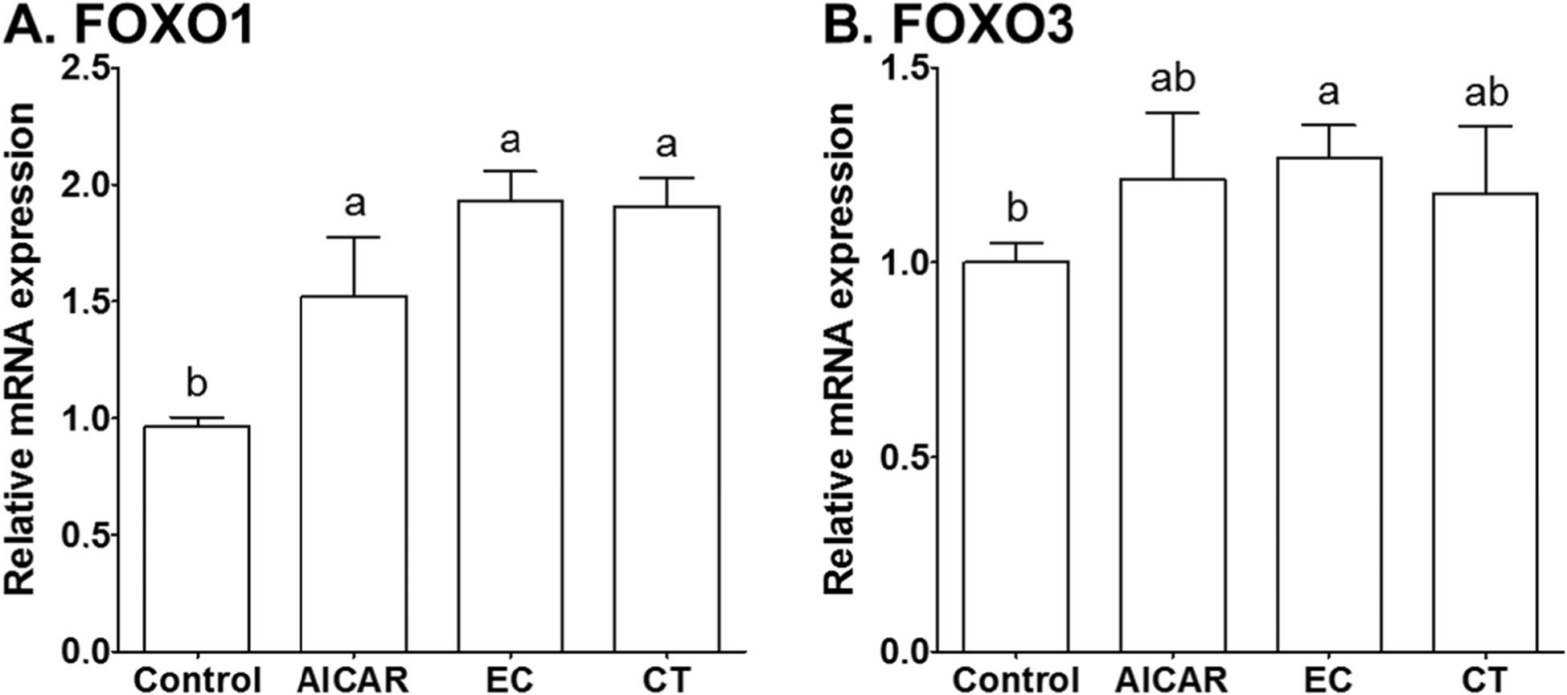
The effects of tannase-treated catechin on the gene expression of (**a**) FOXO1 and (**b**) FOXO3 in C2C12 skeletal muscle cells. Each value represents the mean ± SE. Different letters indicate significant differences at *P <* 0.05 according to Tukey’s test. AICAR: AMPK activator 5-aminoimidazole-4-carboxamide-1-β-D-ribonucleoside; EC: tannase-converted green tea extract containing high epicatechin content; CT: green tea extract

**Fig. 3:**
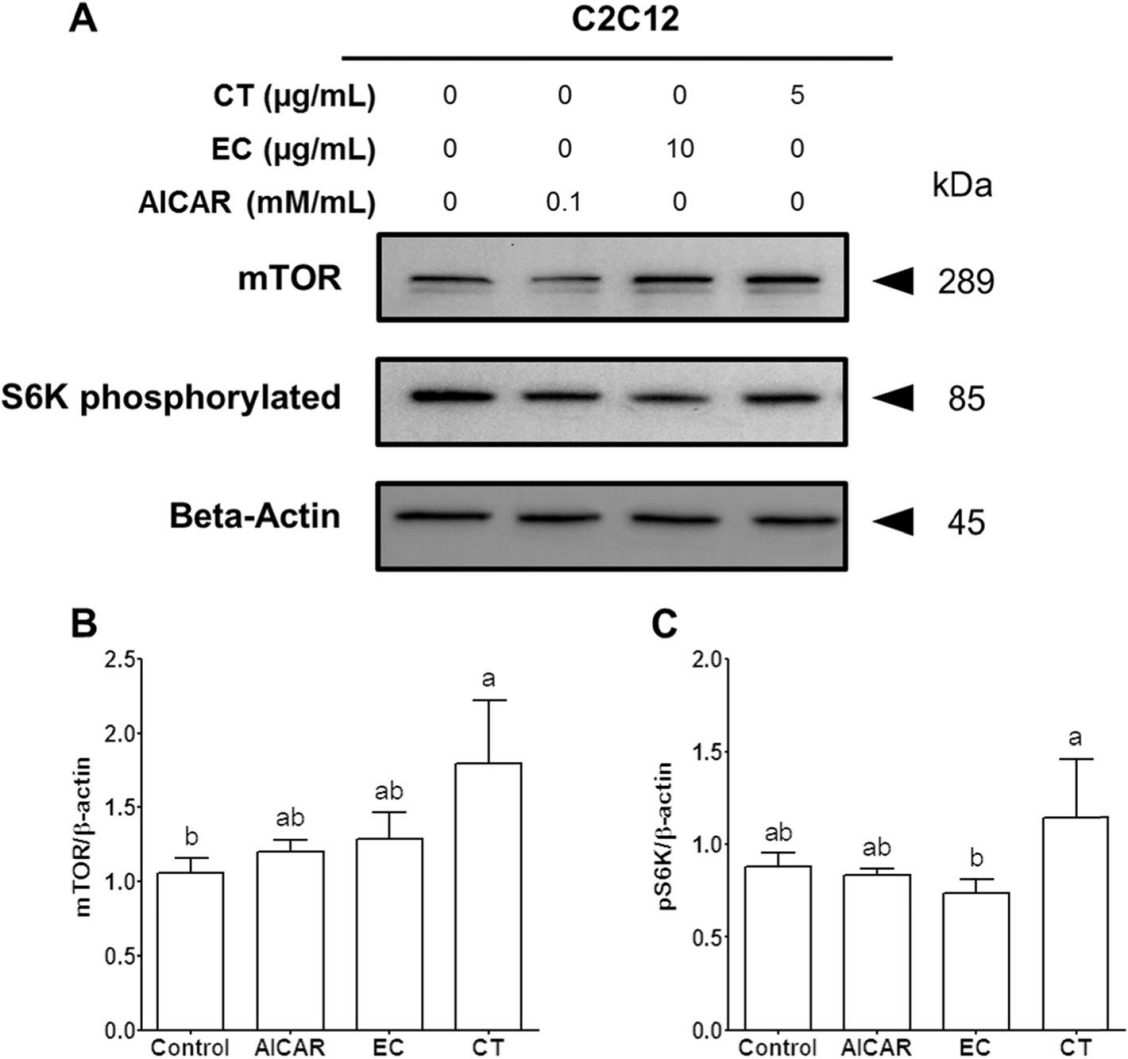
The effects of tannase-treated catechin on levels of mTOR and S6K proteins in C2C12 skeletal muscle cells. Each value represents the mean ± SE. Different letters indicate the significant differences at *P* < 0.05 according to Tukey’s test. AICAR: AMPK activator 5-aminoimidazole-4-carboxamide-1-β-D-ribonucleoside; EC: tannase-converted green tea extract containing a high epicatechin content; CT: green tea extract; mTOR: mammalian target of rapamycin; S6K phosphorylated: p70 S6 kinase

The effects of EC on the gene expression of FOXO1 and FOXO3 in C2C12 cells treated in media with AICAR, EC, and CT for 12 h are shown in Fig. 2. Transcript levels for FOXO1 were significantly higher in the AICAR, EC, and CT groups (Fig. 2a, AICAR: 1.58-fold, *P* < 0.05; EC: 2.00-fold, *P* < 0.05; CT: 1.98-fold, *P* < 0.05) than those in the control group. Additionally, C2C12 cells treated in the EC group showed significantly increased transcript levels for FOXO3, as compared to those observed for the control group (Fig. 2b, EC vs. control, 1.27-fold increase, *P* < 0.05). There was no significant difference in the mRNA levels of FOXO3 in the AICAR and CT groups, as compared to those for the control group (*P* > 0.05). In Fig. 3, this study investigated the effects of EC on FOXO transcription factors in C2C12 cells.

### The effects of Tannase-converted green tea extract on the mTOR/S6K pathway

**Fig. 4:**
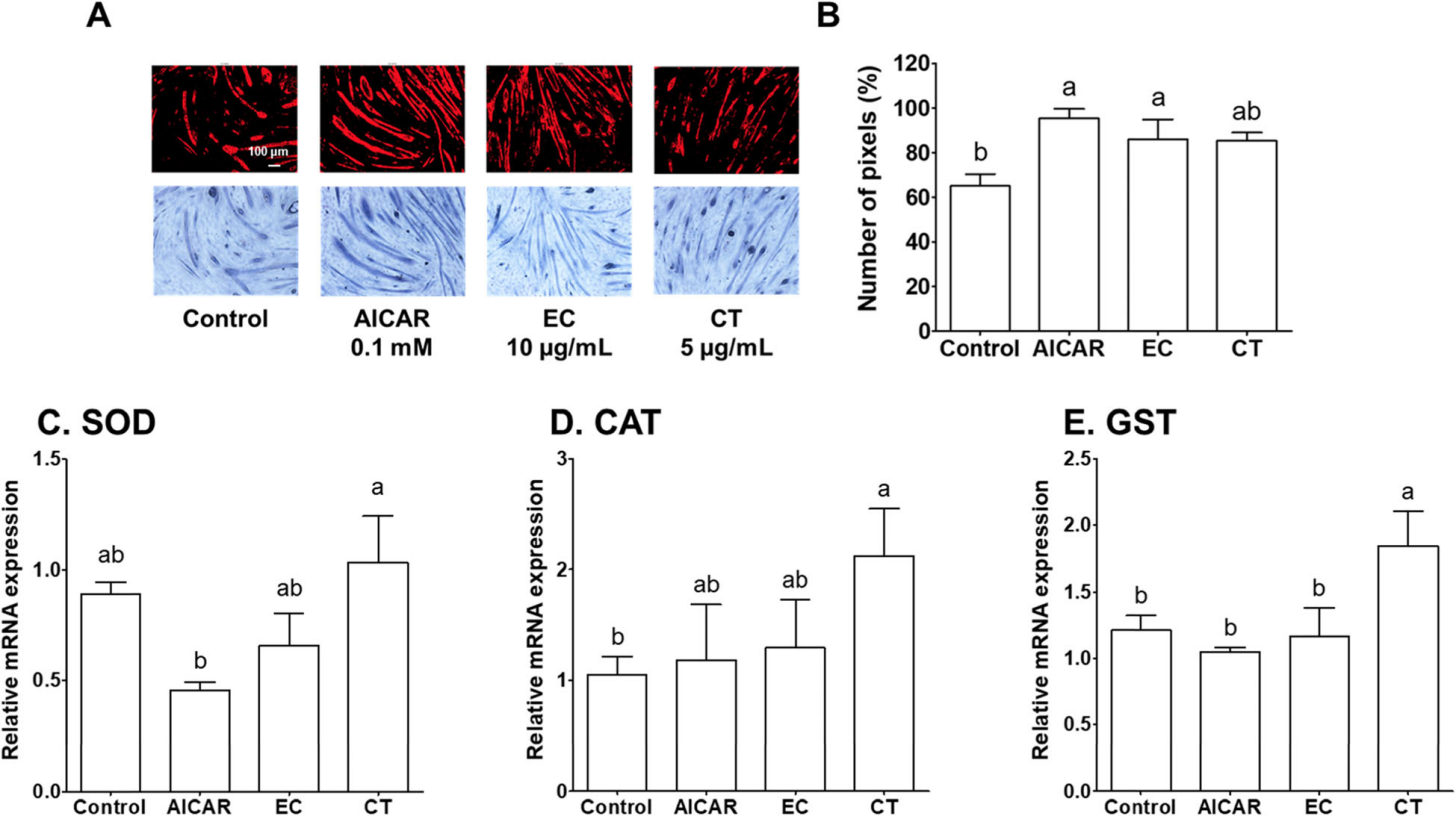
The effects of tannase-treated catechin on (**a** and **b**) myotube formation and the gene expression of (**c**) SOD (**d**) CAT and (**e**) GST in oxidative stress-induced C2C12 skeletal muscle cells. Each value represents the mean ± SE. Different letters indicate significant differences at *P* < 0.05, according to Tukey’s test. AICAR: AMPK activator 5-aminoimidazole-4-carboxamide-1-β-D-ribonucleoside; EC: tannase-converted green tea extract containing a high epicatechin content; CT: green tea extract

The effects of EC on levels of mTOR and pS6K proteins are presented in Fig. 3 and Additional file 3. The mTOR protein levels of C2C12 skeletal muscle cells were significantly increased by treatment with 5 μg/mL CT, as compared to those of the control group (Fig. 4b, CT vs. control, 1.69-fold increase, *P* < 0.05) In addition, the pS6K levels of the CT group were significantly different from those of the EC group (Fig. 3c, CT vs. EC, 1.54-fold increase, *P* < 0.05). However, no significant differences were observed in the levels of mTOR and pS6K proteins in the AICAR and the EC groups, as compared to those of the control group (*P* > 0.05). To better understand the effect of EC on cellular and molecular mechanisms, western blotting was utilized, and the protein levels in the mTOR/S6K pathway were analyzed (Fig. 3).

### The effects of Tannase-converted green tea extract on oxidative stress-induced C2C12 Myogenesis and oxidative stress-related genes

To understand the effects of EC on oxidative stress-induced C2C12 myogenesis, morphological changes due to oxidative stress that were induced by 100 μM of H_2_O_2_ were observed. The morphological changes in the C2C12 cells treated with AICAR, EC, and CT were measured using Giemsa staining, 2 days after exposure to 100 μM H_2_O_2_ (Fig. 4 and Additional file 2). Figure 4b shows that the C2C12 cells exposed to H_2_O_2_ experienced significantly inhibited myogenic differentiation. The myotube density was significantly higher in the AICAR-treated group (vs. control, 1.47-fold increase, *P* < 0.05) and EC group (EC vs. control, 1.32-fold increase, *P* < 0.05). Considering the effects of CT in oxidative stress-induced C2C12 cells, no significant difference in morphological changes were observed, in comparison to those of the control group (*P* > 0.05). The morphological changes induced by H_2_O_2_ have been shown to be caused by oxidative damage, associated with an increase in ROS in cells. Therefore, it was evaluated whether the antioxidant regulation of EC mediates SOD, CAT, and GST gene expression under H_2_O_2_-induced stress. Treatment with CT significantly increased SOD levels approximately 44%, as compared to those of the AICAR-treatment group (Fig. 4c, *P <* 0.05). The mRNA expression of catalase, another antioxidant enzyme, was significantly induced by 50% in the green tea extract group, as compared to that of the control group (Fig. 4c, *P <* 0.05). In addition, green tea extract treatment greatly increased the mRNA level of GST, as compared with that of the control, AICAR, and EC groups (Fig. 4c, *P <* 0.05). These results indicated that AICAR and EC did not affect mRNA levels of antioxidant enzymes, as compared with those of enzymes associated with the morphological change analysis. The data obtained in this study showed that EC effectively suppressed the increase in oxidative stress induced by H_2_O_2_, thereby ameliorating myotube formation (Fig. 4). Additionally, the effects of EC on SOD, CAT, and GST mRNA levels were studied in oxidative stress-induced C2C12 skeletal muscle cells (Fig. 4). However, EC did not have a significant effect on mRNA levels of antioxidant enzymes such as SOD, CAT, and GST. The presence of reactive oxygen species (ROS) has been reported in various muscular disorders, and it is associated with cell injury. These results indicated that EC provided protection against H_2_O_2_-induced oxidative stress in C2C12 cells, which was a result of the radical scavenging effect.

### The effects of Tannase-converted green tea extract on AMPK activity

**Fig. 5:**
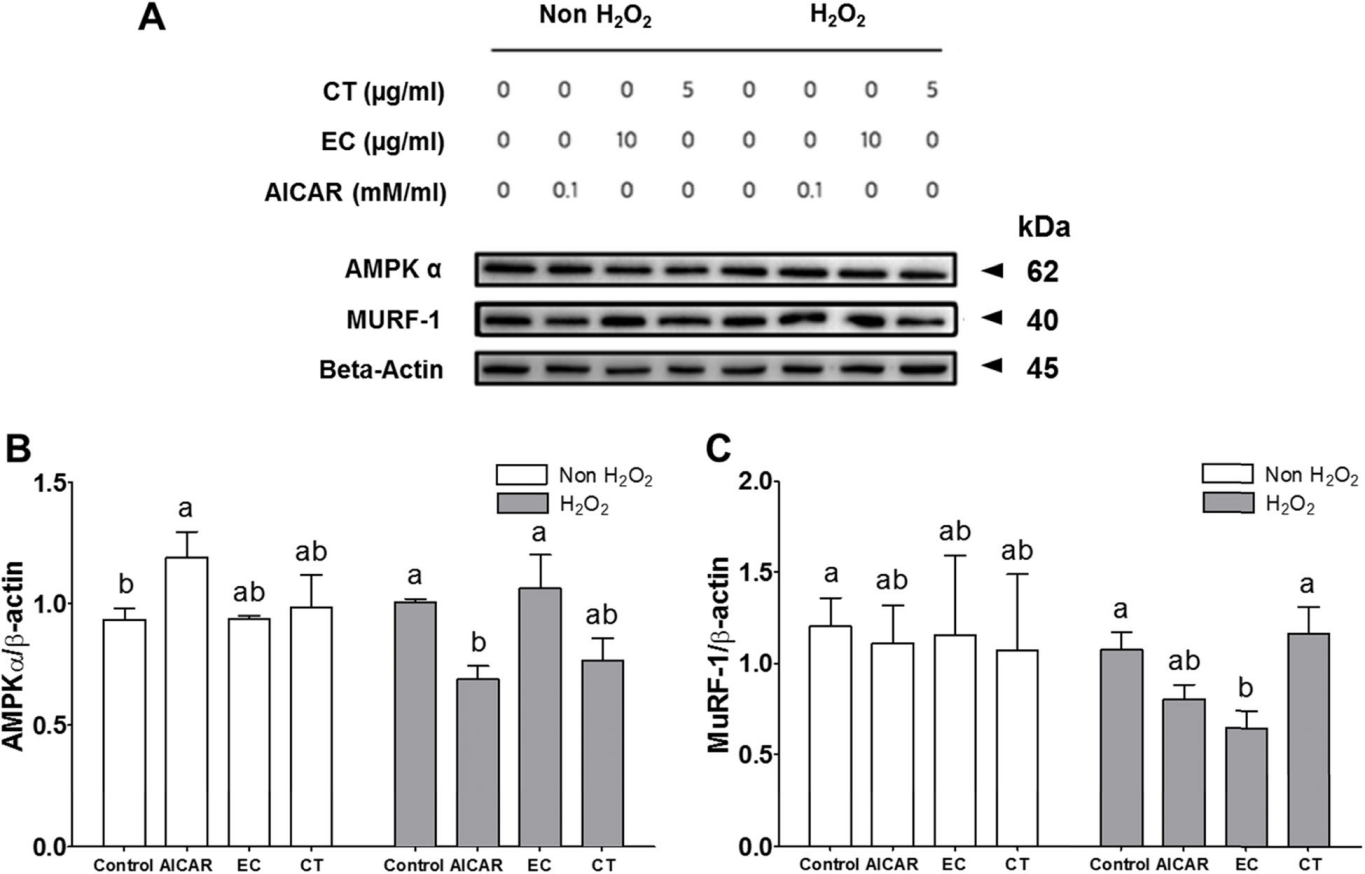
The effects of tannase-treated catechin on levels of AMPK α and MuRF-1 protein in oxidative stress-induced C2C12 skeletal muscle cells. Each value represents the mean ± SE. Different letters indicate significant differences at *P* < 0.05 according to Tukey’s test. AICAR: AMPK activator 5-aminoimidazole-4-carboxamide-1-β-D-ribonucleoside; EC: tannase-converted green tea extract containing high epicatechin content; CT: green tea extract; AMPKα: AMP-activated protein kinase-α; MuRF-1: muscle RING-finger protein-1

To investigate whether the AMPK-dependent mechanism of EC involved a translation process under oxidative stress conditions, the levels of AMPKα and MuRF-1 proteins were determined (Fig. 5 and Additional file 4). Protein levels for AMPKα in normal C2C12 cells of the AICAR group were 1.28-fold higher than that of the control group (*P* < 0.05). In addition, the AMPKα levels in oxidative stress-induced C2C12 cells of the AICAR group were significantly decreased by H_2_O_2_ exposure, as compared to those of the control group and EC group (Fig. 5b, *P <* 0.05). The AMPKα levels of the EC and CT groups were not significantly different in from those of the control group (Fig. 5b, *P* > 0.05). However, the AICAR, EC, and CT groups did not show significant differences in the levels of MuRF-1 proteins, as compared to those of the control group (Fig. 5c). As shown in Fig. 5c, oxidative stress-induced C2C12 cells treated with green tea extract showed significantly different MuRF-1 levels, as compared to those of the control and EC groups (Fig. 5b, *P* > 0.05). As shown in Fig. 5*,* the results found that the treatment of EC increase the levels of AMPKα and MuRF-1 proteins in oxidative stress-induced C2C12 cells. AMPK is the central regulator of metabolism in cells and organisms, and has recently been known to increase myofibrillar protein degradation through the expression of muscle atrophy F-box (MAFbx) and MuRF1 [19]. In addition, the downregulation of atrogin-1 and MuRF1 gene expression, which was involved in the regulation of attenuation of muscle wasting, was investigated.

## Discussion

The four major catechins in green tea extract include approximately 59% EGCG, 19% EGC, 13.6% ECG, and 6.4% EC [20], and green tea also contains GA and other phenolic acids, such as caffeic acid. Baik et al. reported that the addition of green tea extract along with tannase treatment significantly increased the biotransformation of catechins, and pectinase-driven hydrolysis significantly increased interleukin-6 (IL-6) production in macrophages [21]. Dietary polyphenols, including EGCG, resveratrol, and curcumin are associated with the regulation of systemic inflammation and might relieve symptoms of muscle dysfunction [22]. In addition, the AICAR-induced activation of AMPK had an additive effect on glucose transporter-1 (GLUT1) and GLUT4 expression in skeletal muscle, which lead to translocation, which is known to increase the glucose transport response and mitochondrial biogenesis [23].

Lee et al. reported a dose-dependent effect of EC on the protein levels of MHC, MyoD and myogenin, and stimulation of promyogenic signaling pathways, p38 MAPK and Akt, in EC-treated C2C12 myoblasts [24]. In addition, Gutierrz-Salmean et al. proved that EC treatment resulted in a significant increase in the levels of MEF2, Myf5, MyoD, and myogenin in the skeletal muscles of old EC-treated mice (25 months) and the muscle strength in human hands [25]. Experimental evidence found using HepG2 cells and C2C12 skeletal muscle myotubes demonstrated that FOXO transcription factors are sufficient for activating and increasing the levels of a MuRF1 promotor fragment, atrogin-1, and/or MuRF1 mRNA expression [26]. In the skeletal muscle of aged mice, it has been reported that the levels of FOXO3 protein is reduced by 25%, but that there was no change in FOXO1 levels [27]. Phytochemicals, including polyphenols, have been shown to upregulate the functioning of FOXO proteins. The EGCG treatment of rats aged 5 weeks has shown to increase the levels of FOXO3, sirtuin 1, SOD, glutathione peroxidase levels, and their lifespan [28]; the polyphenol curcumin is involved in inhibiting FOXO3 phosphorylation, causing a 2-fold increase in FOXO3-mediated gene expression [29].

AICAR, the positive control, is known to be a direct activator of AMPK that prevents the characteristic increase in muscle protein synthesis that occurs with alterations in mTOR signal transduction [30]. The crosstalk between mTOR/S6K signaling and AMPK is known as the molecular mechanism that controls skeletal muscle mass, and these results have explained both the catabolism and anabolism of skeletal muscle using genetic and pharmacological evidence [31]. Natural products, including EGCG, curcumin, resveratrol, and caffeine have been found to inhibit the mTOR signaling pathway and downstream effector molecules, such as S6K1 [32].

Murakami et al. reported that EC (6.2 μM) had a slightly higher 1,1-diphenyl-2-picrylhydrazyl (DPPH) radical-scavenging activity (EC_50_) than catechin (7.7 μM) [33], and Hong et al. provided specific evidence for the fact that tannase-converted green tea extract has the potential to attenuate UVB-induced oxidative stress in mice skin after the analysis of glutathione (GSH) and hydrogen peroxide levels [34]. In addition, the antioxidant enzyme activity and levels of GSH in C2C12 cells were increased after treatment with polyphenol-rich green tea extract, which thus acted against the oxidative stress caused by mycotoxin citrinin [35].

Flavanol-rich extract and other phenolic compounds are regulated by the genetic expression of atrogin-1 and MuRF1, which alleviated muscle loss and improved impaired myotube formation [36]. In our results, the inhibition of MuRF1 protein levels by EC in oxidative stress-induced C2C12 cells improved impaired myotube formation. Until an approximate age of 40 years, skeletal muscle mass and strength are preserved, but these are reduced to 50% by the age of 80 [37]. Physical activity in the elderly population is limited by sarcopenia, and is associated with a variety of diseases [38]. Therefore, new pharmacological strategies to effectively treat sarcopenia in the elderly can be viewed as a preventive measure. Tannase is an inducible enzyme and decomposes ester bonds in hydrolyzable tannins to produce glucose and gallic acid. It is known that the treatment of green tea with tannase improves the extraction efficiency of polyphenols and increases the radical scavenging ability [14]. Although green tea extract is being investigated in various studies regarding muscle function, recovery, and fibers [39], few studies have evaluated the relationship between skeletal muscle mass and tannase-converted green tea extract. Therefore, this study aimed to investigate the effects of tannase-converted green tea extract with a high EC, EGC, and gallic acid (GA) content on cellular morphological changes and intercellular signaling pathways, using well-characterized models of normal C2C12 and oxidative stress-induced C2C12 skeletal muscle cells.

## Conclusions

In conclusion, as compared to CT, the green tea extract converted to hydrolyzed tannase contributed to a greater improvement in myotube formation and protective properties against H_2_O_2_-induced oxidative stress in C2C12 cells. The effects of EC with a high EC, EGC, and GA content were demonstrated by an improvement in the regulation of muscle regulatory factors, transcription factors, and the mTOR/S6K pathway, as well as by Giemsa staining analysis. The properties of EC are considered to be a result of the radical scavenging ability and downregulation of MuRF1 protein levels in oxidative stress-induced cells. Taken together, these results suggest that EC with a high EC, EGC, and GA content can be used as a supplement for alleviating muscle loss in C2C12 skeletal muscle cells. Moreover, the outcomes of this study are expected to shed light on cellular and molecular mechanisms for further understanding the functional and pharmacological properties of botanical extracts, their enzymatic hydrolysis, and their therapeutic potentials for sarcopenia. Further in vivo studies for the myostatin and follystatin signaling pathways are necessary, irrespective of whether the myogenin expression observed in this study was caused because of them or by inflammatory cytokine pathway regulation. In summary, this study supports that tannase-converted green tea extract is the principal material that modulates intracellular signaling pathways to prevent or treat muscle atrophy.

## Supplementary information

**Additional file 1: Figure S1** Raw data for Fig. 1.

**Additional file 2: Figure S2** Raw data for Fig. 4.

**Additional file 3: Figure S3** Raw data for Fig. 3.

**Additional file 4: Figure S4** Raw data for Fig. 5.

## Data Availability

The dataset generated during the present study is available upon reasonable request to the author (Prof. Yooheon Park).

## Abbreviations

AICAR: AMPK activator 5-aminoimidazole-4-carboxamide-1-β-D-ribonucleoside
AMPKα: AMP-activated protein kinase-α
IL: Interleukin
MAFbx: Muscle atrophy F-box
MuRF-1: Muscle RING-finger protein-1
ROS: Reactive oxygen species
