# Supplementary figures and images for: Effects of tannase-converted green tea extract on skeletal muscle development
Source: BMC Complement Med Ther. 2020 Feb 11;20:47. doi: 10.1186/s12906-020-2827-7 (PMC7076851; doi:10.1186/s12906-020-2827-7)

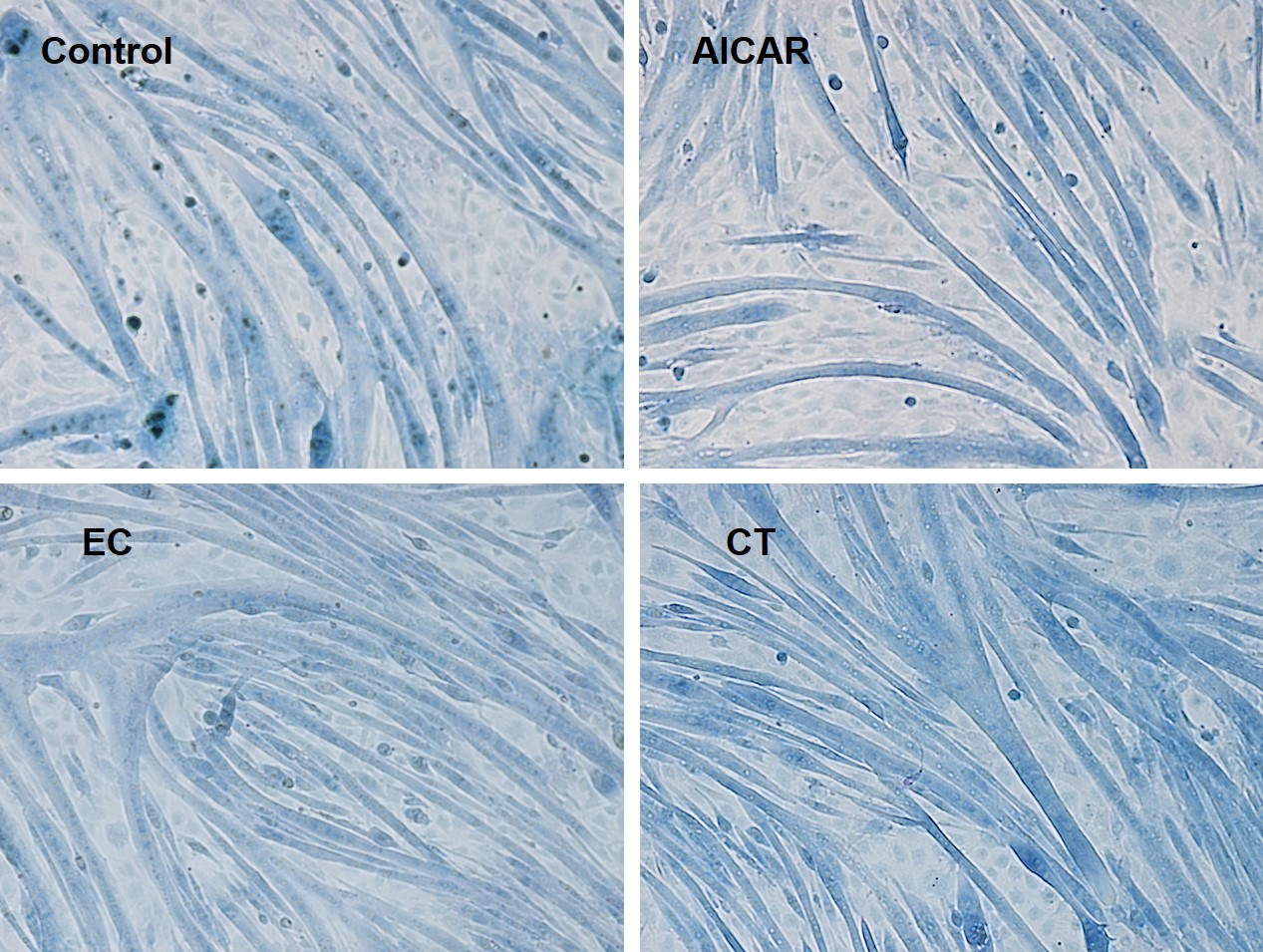

Supplement: Supplementary file 1 — Additional file 1: Figure S1 Raw data for Fig. 1. [file 12906_2020_2827_MOESM1_ESM.jpg]

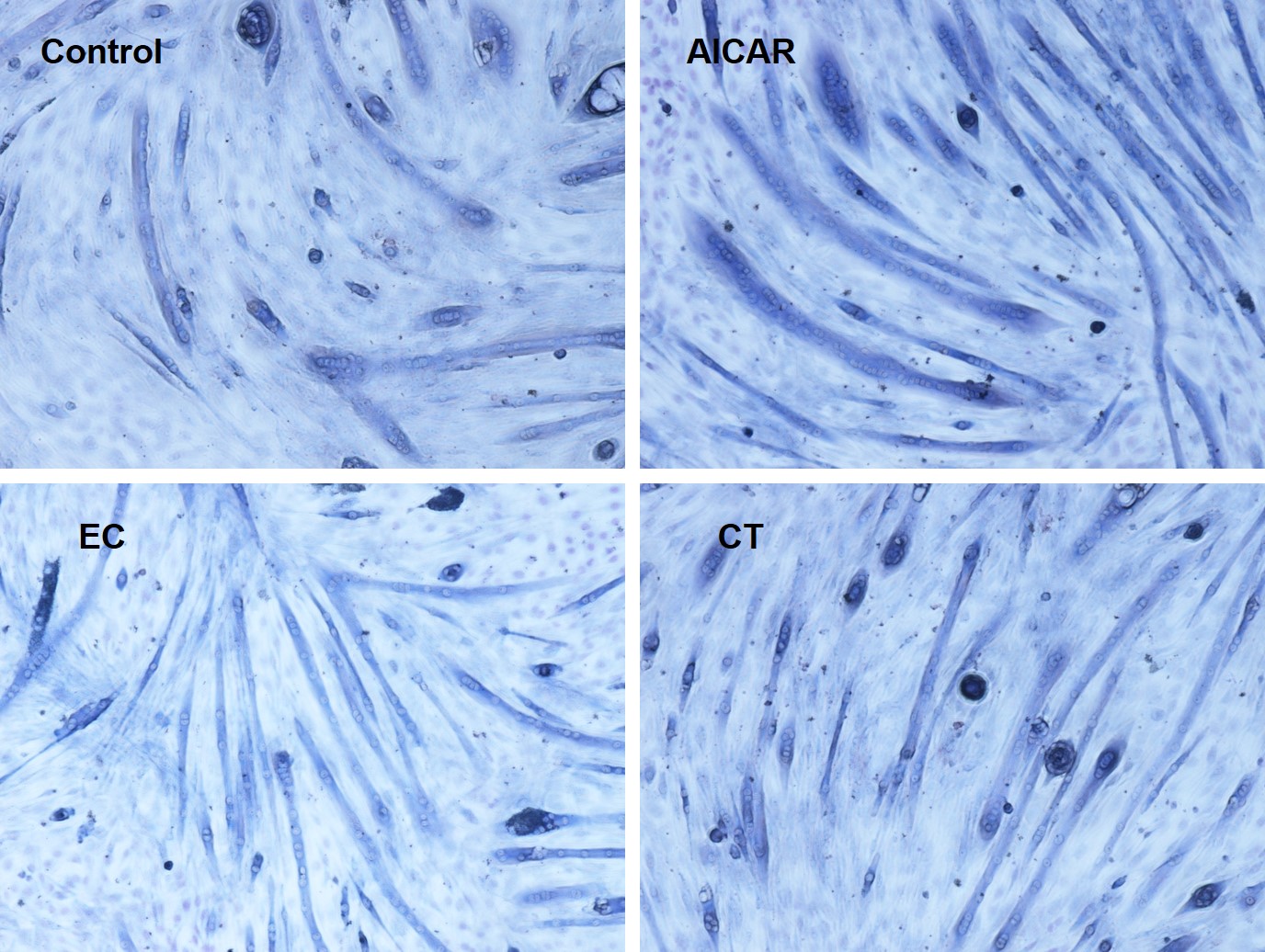

Supplement: Supplementary file 2 — Additional file 2: Figure S2 Raw data for Fig. 4. [file 12906_2020_2827_MOESM2_ESM.jpg]

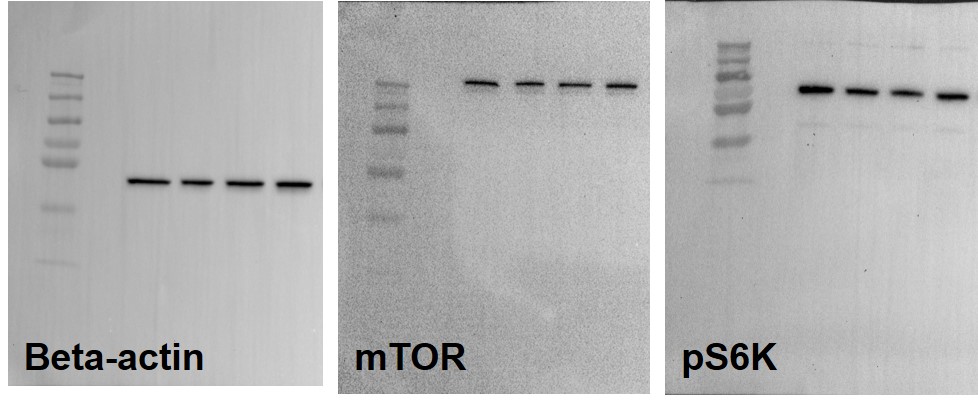

Supplement: Supplementary file 3 — Additional file 3: Figure S3 Raw data for Fig. 3. [file 12906_2020_2827_MOESM3_ESM.jpg]

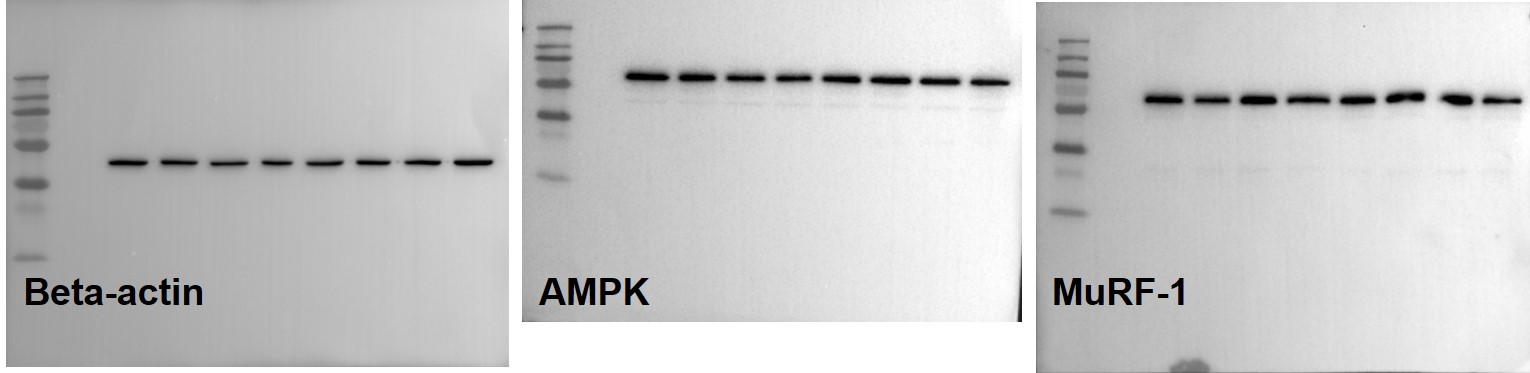

Supplement: Supplementary file 4 — Additional file 4: Figure S4 Raw data for Fig. 5. [file 12906_2020_2827_MOESM4_ESM.jpg]
